# Estimating yield gaps at the cropping system level^☆^

**DOI:** 10.1016/j.fcr.2017.02.008

**Published:** 2017-05

**Authors:** Nicolas Guilpart, Patricio Grassini, Victor O. Sadras, Jagadish Timsina, Kenneth G. Cassman

**Affiliations:** a Department of Agronomy and Horticulture, University of Nebraska-Lincoln, Lincoln, NE, USA; b AgroParisTech, UMR Agronomie 211 INRA AgroParisTech Université Paris-Saclay, F-78850 Thiverval-Grignon, France; c South Australian Research and Development Institute, Waite Campus, Adelaide 5001, Australia; d Faculty of Veterinary and Agricultural Sciences, University of Melbourne, Victoria 3010, Australia

**Keywords:** Yield potential, Yield gap, Cropping system, Rice, Maize, Bangladesh

## Abstract

Yield gap analyses of individual crops have been used to estimate opportunities for increasing crop production at local to global scales, thus providing information crucial to food security. However, increases in crop production can also be achieved by improving cropping system yield through modification of spatial and temporal arrangement of individual crops. In this paper we define the cropping system yield potential as the output from the combination of crops that gives the highest energy yield per unit of land and time, and the cropping system yield gap as the difference between actual energy yield of an existing cropping system and the cropping system yield potential. Then, we provide a framework to identify alternative cropping systems which can be evaluated against the current ones. A proof-of-concept is provided with irrigated rice-maize systems at four locations in Bangladesh that represent a range of climatic conditions in that country. The proposed framework identified (i) realistic alternative cropping systems at each location, and (ii) two locations where expected improvements in crop production from changes in cropping intensity (number of crops per year) were 43% to 64% higher than from improving the management of individual crops within the current cropping systems. The proposed framework provides a tool to help assess food production capacity of new systems (*e.g.* with increased cropping intensity) arising from climate change, and assess resource requirements (water and N) and associated environmental footprint per unit of land and production of these new systems. By expanding yield gap analysis from individual crops to the cropping system level and applying it to new systems, this framework could also be helpful to bridge the gap between yield gap analysis and cropping/farming system design.

## Highlights

• Previous yield gap analyses have focused on individual crops.
• We developed a framework to estimate cropping system yield potential and yield gap.
• A proof-of-concept is provided with a case study on rice-maize cropping systems in Bangladesh.
• The proposed framework identified opportunities to increase cropping system annual yield.

## 1 Introduction

Food security must account for opportunities to increase production against projected changes in demand associated with population growth and changing diets, need to reduce the environmental footprint of agriculture, and limited availability of land suitable for crop production (Cassman et al., 2003, Godfray et al., 2010, Foley et al., 2011). Yield gap is the difference between yield achieved by farmers and potential or water-limited potential yield (*i.e.* rainfed yield potential). Analysis of yield gaps helps identify opportunities to improve crop yield and assess food security scenarios (Van Ittersum et al., 2013, Fischer, 2015, Timsina et al., 2016, Van Ittersum et al., 2016). Yield gap analysis has been performed for a number of staple food crops in different regions (Liu et al., 2012, Van Rees et al., 2014, Grassini et al., 2015b, Van Oort et al., 2015, Marin et al., 2016, Timsina et al., 2016) at both local and global scales (Affholder et al., 2013, Mueller et al., 2013), but in all these studies the focus has been on individual crops.

However, important improvements in productivity also are likely to come from innovations at the cropping or farming system levels (Rodriguez and Sadras, 2011). In temperate rainfed agriculture, where rainfall and temperature constrain the system to a single crop per year, crop rotations are particularly relevant to farm-level production (Angus et al., 2015, Farmaha et al., 2016). In environments with a longer growing season where multiple cropping is feasible, capture of resources and yield are often improved through the processes of facilitation and niche differentiation in time and space (Brooker et al., 2014, Gaba et al., 2014, Li et al., 2007, Malézieux et al., 2009). In Argentina for example, greater cropping intensity (number of crops in a 12-month period) increased efficiency in use of incident solar radiation, and total yield of the cropping system (Andrade et al., 2015).

Improvements at the cropping system (CS) level are often associated with lower yields of individual crops that together give greater total yield than from maximizing yield of a single crop (Evans, 1993, Timsina and Connor, 2001, Egli, 2008, Fletcher et al., 2011, Hochman et al., 2014). This trade-off between individual crop and system-level yield has been reported in many, diverse production systems (Table 1). Sometimes, the yield increase at CS level may occur at the expense of greater yield variability for one of the crops within the sequence, for example, for late-sown soybean in a wheat–soybean annual double-crop system in Argentina (Monzon et al., 2007). Therefore, a framework is needed to estimate yield gaps at the CS level that accounts for the time dimension (*i.e.*, yield expressed in kg ha^−1^ yr^−1^) and yield stability (*e.g.*, inter-annual coefficient of variation) as influenced by the spatial and temporal arrangement of crops. This is particularly relevant in the context of a global increase in cropping intensity in recent decades (Ray and Foley, 2013, Wu et al., 2015).

A number of challenges have been identified to assess yield gap at the CS level. First, despite few attempts to quantify yield gaps at the system level (Liang et al., 2011, Hochman et al., 2014, Henderson et al., 2016), a robust definition of CS yield potential is lacking. Second, it would be useful to identify the “best” CS to benchmark current systems, but the notion of an “optimal” CS is inconsistent with theory and evidence (Sadras and Denison, 2016). For example, even in intensive, high-yield CSs, average farm yield is 15–25% below yield potential (Van Wart et al., 2013). This is partially related to farmers’ decisions to satisfy multiple and often opposing objectives (*e.g.*, high profit, high resource-use efficiency, low risk, minimal environmental impact) within the social, political, economic, and environmental constraints that confront their operations (Simon, 1955, De Wit, 1992, Sadras and Denison, 2016). Therefore, to disentangle the confounding effects of biophysical and socioeconomic factors on CS yield, we propose a framework to estimate CS yield gap from a strictly biophysical perspective as a first step in the evaluation process that would also consider socioeconomic considerations as well.

Our first objective is to define the CS yield potential necessary to estimate the associated yield gap. Then, we develop an operational framework to estimate the CS yield potential and the associated yield gap at different spatial scales (*e.g.*, location, region, country). We apply this framework to a case study on irrigated rice-maize CSs in Bangladesh to test the hypothesis that closing CS yield gaps can, in some cases, give higher productivity gains than closing the yield gaps of individual crops. Finally, owing to environmental concerns about large input requirements of high-yielding agriculture, especially water (Famiglietti, 2014, Siebert et al., 2010) and nitrogen (Cassman et al., 2002, Ladha et al., 2016), we evaluate the amount of water and nitrogen (N) needed to realize the expected yield potential of the identified alternative cropping systems with greater yield potential.

## 2 Methods

### 2.1 Conceptual framework

#### 2.1.1 Cropping system yield potential and associated yield gap

For individual crops, yield potential (Yp) is defined as the yield of an adapted cultivar when grown with water and nutrients non-limiting and biotic stresses (weeds, pests, and diseases) effectively controlled (Evans, 1993). Similarly, for a given CS (noted CS*_i_*) the yield potential (noted CSYp *_i_*) can be defined as the sum of yields of all crops in this CS, when crop growth is not limited by water, nutrients, or biotic stresses. To compare systems that include crop species with different grain composition (*e.g.*, cereal and oilseed crops), and systems with different cropping intensities (*e.g.* one *vs* three crops per year) and temporal arrangements of crops (*e.g.* sole crop *vs* intercropping), this metric needs to be expressed in energy per unit land and time (GJ ha^−1^ yr^−1^), calculated as the product of three factors: harvested yield mass, dry matter content, and energy content. Over a period of *m* years during which *n* crops are grown, yield potential (CSYp *_i_*) and actual yield (CSYa *_i_*) of CS*i* can be calculated as:

(1) $$CSYp_{i}=\frac{1}{m}\overset{n}{\underset{j=1}{\sum }}Yp_{i,j}$$

(2) $$CSYa_{i}=\frac{1}{m}\overset{n}{\underset{j=1}{\sum }}Ya_{i,j}$$

where Yp *_i,j_* and Ya *_i,j_* are potential and actual yields of crop *j* in CS*i*, expressed in GJ ha^−1^ yr^−1^. These definitions apply to any CS.

Then, (absolute) CS yield potential (CSYp^*^) can be defined as the output from the combination of crops that gives the highest energy return per unit of land and time, and can be calculated as follows:

(3) $$CSYp^{*}=\underset{i\in I}{\sup}\left(CSYp_{i}\right)$$

where *I* is the set of all possible CSs at the location of interest.

The CS that achieves CSYp^*^ will be noted CS^*^. Similarly to Yp for individual crops, CSYp^*^ is location specific because of the climate. Although important, CSYp^*^ is not sufficient to evaluate and compare the performances of CSs due to the many (and often conflicting) productivity, environmental and economic goals. For example, economic net return ($ ha^−1^ yr^−1^), downside risk (*e.g.*, probability of negative net return or crop failure), and environmental impact (*e.g.*, nutrient and pesticide leaching) are important factors driving choice of CS. However, these metrics would vary considerably with time and regions due to variation in commodity prices, input costs, environmental concerns and regulations, and farmer’s risk attitude. Having recognized this limitation, our study focuses on a framework to estimate CSYp^*^ and its variability, which can be quantified by the inter-annual coefficient of variation (CV). Such an assessment represents an essential first step to extend yield gap analysis from individual crop to cropping system.

Hereafter, the CS yield gap of an existing CS (noted CS*_i_*) will be noted CSYg *_i_* and is defined as:

(4) $$CSYg_{i}=CSYp^{*}-CSYa_{i}$$

**Fig. 1:**
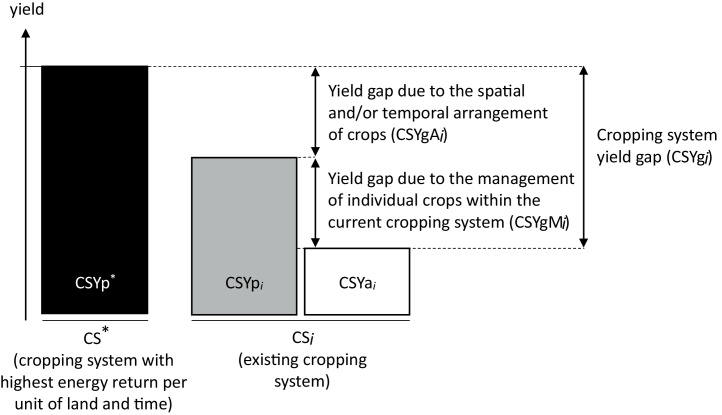
Conceptual framework representing the cropping system yield gap and its components. CS*_i_* is an existing cropping system. CSYp *_i_* is the yield potential of CS*_i_* as defined in Eq. (1). CSYa *_i_* is the actual yield of CS*_i_* as defined in Eq. (2). CS^*^ is a new cropping system, defined as the system with highest energy return per unit of land and time (Eq. 3). CSYp^*^: yield potential of CS^*^, also called cropping system yield potential. The subscript *i* denotes that in a given location there may be many existing cropping systems (*i* can take many values), while there is only one CS^*^. See main text Section 2.1 for definitions. To compare systems that include crop species with different grain composition, cropping intensities, and temporal arrangements of crops, all yields are expressed in energy per unit land and time.

Then, CSYg *_i_* can be disaggregated into two components (Fig. 1): the yield gap due to the management of individual crops within the current CS*_i_* (CSYgM*_i_*) and the yield gap due to the spatial and/or temporal crop arrangement in current CS*_i_* relative to CS^*^ (CSYgA*_i_*):

(5) $$CSYg_{i}=CSYgM_{i}+CSYgA_{i}$$

The relative importance of CSYgM and CSYgA provides insight about opportunities to improve yield by closing yield gaps of individual crops within current CS, by improving the spatial and/or temporal arrangement of crops, or a combination of both options. This paper focuses on crop-based systems, but the same definitions of yield potential and associated yield gap could be used at the farming system level if other production enterprises^1^ were included, such as livestock (*e.g.*, Van der Linden et al., 2015).

#### 2.1.2 Framework to identify alternative cropping systems

**Fig. 2:**
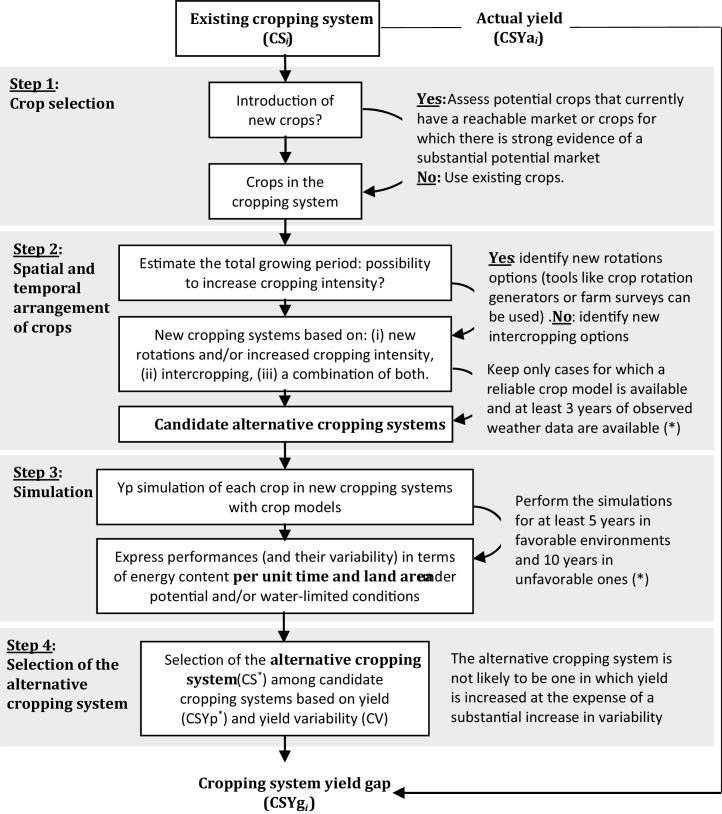
Framework to estimate yield gap at the cropping system level. (*) Readers are referred to Grassini et al. (2015a) for further details about yield gap analysis for single crops.

Estimating CSYp^*^ as proposed in Eq. (3) raises two challenges: (i) identification of *I* the set of all possible CSs at the location of interest, and (ii) calculation of CSYp for all CSs of *I*. Indeed, the number of possible crops and CSs may be large in some cases, thus limiting our ability to quantitatively evaluate all of them, even by simulation with crop models. More importantly, there are biological and agronomic constraints to optimize CSs (Sadras and Denison, 2016). Therefore, rather than searching for an optimum CS which may not exist, we propose a framework to identify relevant alternative CSs against which the current CSs could be compared (Fig. 2), allowing for a realistic estimation of CSYp^*^ and CS^*^.

The proposed framework includes four steps (Fig. 2): (i) selection of crops, (ii) definition of their spatial and temporal arrangement, (iii) simulation of the candidate alternative CSs, and (iv) selection of the most appropriate CS as the alternative CS (CS^*^) based on annual energy yield (CSYp^*^) and its stability. The framework is based on three principles: (i) new crops, if introduced, should either have a reachable market or there is evidence of a substantial potential market; the purpose of this condition is to emphasize that focus should be put on the most promising alternative CSs rather than all possible CSs; (ii) the option of increased cropping intensity depends on the total growing period as constrained by photoperiod, temperature, radiation, water supply, and the range of maturities of selected crop species, and (iii) the analysis must account for the downside risk of the alternative CS (Monjardino et al., 2015). Enabling tools include reliable crop models to simulate the CSs of interest, a long-term weather database to quantify climate-driven processes governing system performances and associated risks, a soil database to retrieve functional soil properties that influence soil water storage and crop water uptake, and information on current management practices (Grassini et al., 2015a). The proposed framework relies on several assumptions (Table 2). For instance, effects of previous crop(s) on yields due to residual soil N and water, and greater incidence of biotic factors in high-yield crops are not taken into account. Indeed, according to the definition of yield potential (Evans, 1993), effects of previous crop(s) mediated by water, nutrients, soil structure, pests and diseases do not affect yield potential because they are assumed to be overcome by use of optimal crop and soil management practices that eliminates all constraints other than climate.

### 2.2 Case study: irrigated rice-maize cropping systems in Bangladesh

#### 2.2.1 Overview

Cropping systems in Bangladesh have high cropping intensity and diversity of species. Current systems are predominantly rice-based, involving one or more crops per year in annual sequences, with an average cropping intensity approaching two crops per year (Timsina et al., 2016). Irrigated rice-maize systems in Bangladesh were selected as a case study for three reasons. First, the sub-tropical climate and widespread access to water for irrigation allows high cropping intensities. Double cropping is widespread, *e.g.* rice-rice, rice-wheat and rice-maize (Timsina et al., 2010, Timsina et al., 2011) and three rice crops per year are feasible in some regions (Gumma et al., 2014, Timsina et al., 2016). Second, robust crop simulation models are available for maize (Hybrid-Maize, Yang et al., 2004), wheat (WOFOST, Supit et al., 1994), and rice (ORYZA, Bouman et al., 2001), which have already been locally evaluated on their performance to estimate yield potential and applied to estimate yield gaps of individual crops (Timsina et al., 2016). Third, relevant databases are available through the Global Yield Gap Atlas, including long-term weather, cropping systems, soil, and actual farm yield data (www.yieldgap.org/bangladesh).

**Fig. 3:**
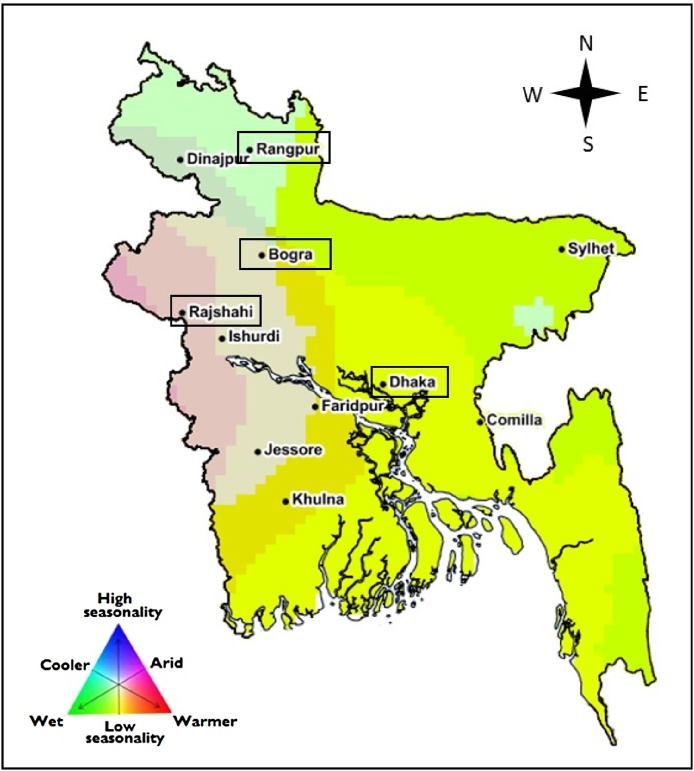
Climate zones in Bangladesh and the four selected locations: Bogra, Dhaka, Rajshahi, and Rangpur. Climatic zones are based on a matrix of three categorical variables (growing degree days, aridity index, and temperature seasonality) as described in Van Wart et al. (2013).

Source: *Global Yield Gap Atlas* (www.yieldgap.org).

Four sites were selected from the Global Yield Gap Atlas: Bogra, Dhaka, Rajshahi, and Rangpur (Fig. 3). These sites capture the range of climate, soils and management practices across major crop producing regions in the country. Three cropping seasons were considered: the rainy season *aman* (rice) or *kharif-II* (maize) from June-July to September-October, the dry season *boro* (rice) or *rabi* (maize) from October-November to February-March and the *aus* (rice) or *kharif-I* (maize) season from March-April to May-June. Monthly averages of main climatic variables are shown in Fig. S1. Dominant current cropping systems involving rice, wheat and maize were retrieved from the Global Yield Gap Atlas (Tables S1 and S2). In this paper, we focus on rice-maize cropping systems because (i) demand for maize used in livestock production is rising rapidly and area of rice-maize systems is expanding in South Asia and especially in Bangladesh (Timsina et al., 2010, Timsina et al., 2011), and (ii) wheat harvested area in Bangladesh has strongly decreased during the past 15 years with a 50% reduction from 2000 to 2014 (FAO, 2016). This led to a set of five current CSs, whose relative proportions (on an area basis) were taken from the Global Yield Gap Atlas for each location in this study (Table 3).

CSYg of each current system was estimated in three steps. First, actual (Ya) and potential yield (Yp) of individual crops within current systems were retrieved from the Global Yield Gap Atlas, and CSYa and CSYp of current systems were calculated according to Eqs. (1) and (2). Data and methods used in the Global Yield Gap Atlas to estimate individual crops Yp are available at: http://www.yieldgap.org/bangladesh. Note that crop models used in this paper are the same as those used in the Global Yield Gap Atlas, ensuring consistency between simulated yield potential reported here and those in the Atlas. Second, the CS^*^ at each location was identified following Fig. 2 framework (see Section 2.2.2 for details). Third, CSYg *_i_* was calculated as the difference between CSYp^*^ and CSYa *_i_* for each current CS*_i_* (Fig. 1 and Eq. (3)). Following Van Bussel et al. (2015), single estimates of CSYp, CSYa, and CSYg at each location were obtained by weighting CSYp *_i_*, CSYa *_i_*, and CSYg *_i_* of current CSs by their relative crop area in the region surrounding each of the four sites where CSYg was evaluated (Table 3).

#### 2.2.2 Identification and simulation of alternative cropping systems

At each location, the CS^*^ was identified following the four steps of the framework in Fig. 2. We focused on rice and maize so that candidate alternative CSs only involved these crops, both of which have large existing markets. Intercropping was not considered because, except for small areas where maize is intercropped with potato, both rice and maize are not intercropped in Bangladesh. Identification of candidates alternative rice-maize CSs was based on simulations using: ORYZA(v3) for rice (Bouman et al., 2001) and Hybrid-Maize for maize (Yang et al., 2004). We simulated four generic rice varieties (extra-short, short, intermediate, late growth duration) and four maize hybrid maturity groups at each location. These maturity ranges represent commercially available rice and maize germplasm in Bangladesh and are captured in the phenological parameters presented in Table S3. Twenty-four sowing dates (for maize) and transplanting dates (for rice), spaced at 15-d intervals, were simulated, with one simulation per variety and sowing/transplanting date over 14 years (1992–2005) at each location. Plant density was set to 75 plants m^−2^ for rice and 8 plants m^−2^ for maize to reflect current practices in intensive, high-yield irrigated rice and maize systems in favorable environments (Yoshida, 1981, Huang et al., 2013, Grassini et al., 2009). For both crops, simulations assumed that crop yield was not limited by water, nutrients, or biotic stresses (Table 2). Simulations were run independently with ORYZA(v3) and Hybrid-Maize so that the models were not coupled.

Grain yield (t ha^−1^) was calculated at 15.5% moisture for maize and 14% moisture for rice, which correspond to commercial standards. Energy yield (GJ ha^−1^) was calculated as the product of grain yield and grain energy content, for which we used the following values: 1440 kJ per 100 g of rice at 14% moisture, and 1480 kJ per 100 g of maize at 15.5% moisture (USDA National Nutrient Database). For each sowing/transplanting date by crop cultivar combination, we estimated the average simulated Yp and the temporal stability of simulated Yp (quantified by the coefficient of variation) over the 1992–2005 period. All possible crop sequences (with one, two or three crops per year) were evaluated by: (i) creating all crop sequences by permutation of individual crops (rice and/or maize), (ii) discarding crop sequences for which two crop cycles overlapped, or with less than 3 weeks of fallow period between physiological maturity of a preceding crop and sowing or transplanting of a following crop to allow enough time for harvest and land preparation (Krupnik et al., 2015), (iii) calculating CSYp and its CV of each possible crop sequence according to Eq. (1), both in GJ ha^−1^ yr^−1^ and in t ha^−1^ yr^−1^, (iv) discarding systems having a CV in yield higher than 10%, which is relatively high for irrigated crop production (Grassini et al., 2014). Finally, at each location CS^*^ was selected as the alternative CS with the highest CSYp (Eq. 3).

#### 2.2.3 Resource capture

Improvement in supply and capture of water and N are major drivers of yield improvement on historic time scales (Sinclair and Rufty, 2012). Further, massive expansion of water withdrawals for irrigation have lowered groundwater levels in many areas of Bangladesh (Kirby et al., 2015). Therefore, we estimated the requirements of water and N to achieve CSYp of current systems and CSYp^*^. Annual crop evapotranspiration was obtained from the simulations. As models were run under potential conditions, N budget was not simulated, and hence annual crop N uptake was calculated using the crop yield (kg ha^−1^) *versus* crop N uptake (kg N ha^−1^) relationship reported by Cassman et al. (2002) for rice and maize:

(6) $$riceyield=-1573+643\times {\left(N_{uptake}\right)}^{0.5}$$

(7) $$maizeyield=-3710+995\times {\left(N_{uptake}\right)}^{0.5}$$

Note that we considered these relationship to hold under potential conditions, which is a reasonable assumption as data used for their calibration came mostly from well managed maize and rice crops (Cassman et al., 2002). No attempt was made to account for the variation in grain protein in the calculation of N uptake (Sadras, 2006, Gastal et al., 2014). As a complement for the analysis of water and N, we also estimated the simulated fraction of annual photosynthetically active radiation (PAR) intercepted by the crops.

## 3 Results

### 3.1 Alternative cropping systems and cropping system yield potential

**Fig. 4:**
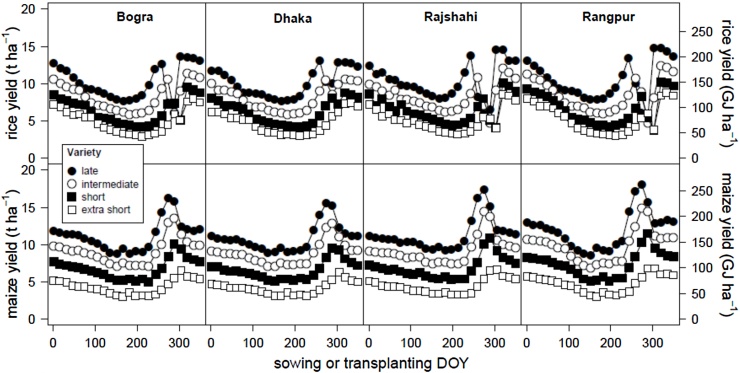
Simulated yield (average 1992–2005) of rice (top row) and maize (bottom row) crops in four locations as related to variety maturity and sowing or transplanting date. Simulations assumed no limitations by water, nutrients, pests and diseases. Yields are expressed in t ha^−1^of grain at standard moisture contents (left axis), and GJ ha^−1^ of energy content of grain (right axis). DOY: day of year.

Fig. 4 shows average simulated Yp (under no limitation by water, nutrients, pests and diseases) of rice and maize as a function of sowing (maize) and transplanting date (rice) and crop maturity group by location. As expected, varieties with a longer growing cycle had greater yield potential. At all locations, largest Yp was obtained with a late variety sown on October 1st for maize and when transplanting occurred in November 15th for rice, and ranged from 220 to 270 GJ ha^−1^ (15–18 t ha^−1^) for maize and from 190 to 210 GJ ha^−1^ (13–14.5 t ha^−1^) for rice. For both crops, highest Yp was associated with relatively high yield stability as indicated by a CV ranging from 3 to 10% across all locations (Fig. S2). Simulated rice Yp showed a marked decrease (Fig. 4) and its CV a marked increase (Fig. S2) when transplanting occurred on late October (around DOY 300). Analysis of simulations revealed that this was due to an increased risk of cold injury during the period from panicle initiation to flowering in January-February, the coldest period of the year (*data not shown*).

CS^*^ are presented in Table 4. In all locations, the CS^*^ differed from the current dominant CSs and was an annual triple-crop system with *kharif-II* maize, *rabi* maize and *aus* rice, except in Rajshahi where the CS^*^ crop sequence used rice (*aman*), maize (*rabi*) and maize (*kharif-I*). CSYp^*^ ranged from 480 GJ ha^−1^ yr^−1^ (32.5 t ha^−1^ yr^−1^) in Rajshahi to 504 GJ ha^−1^ yr^−1^ (34.2 t ha^−1^ yr^−1^) in Rangpur, with high yield stability as indicated by a CV <8% at all sites (Table 4). Not surprisingly, CSYp^*^ was 24% to 270% greater than CSYp of existing systems across all locations because CS^*^ had three crops per year, including two high-yielding hybrid maize crops, compared to existing systems that typically had one or two crops per year with, at best, one maize crop.

### 3.2 Cropping system yield gap and its components

**Fig. 5:**
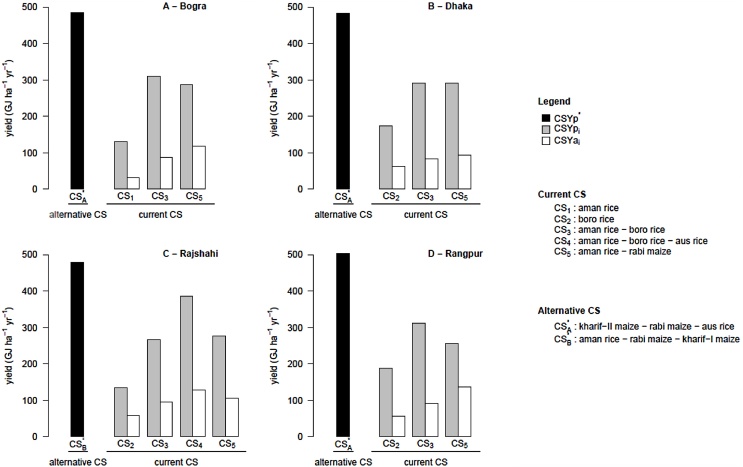
Cropping system yield potential (black bars), yield potential of current cropping systems (grey bars) and actual yield of current cropping systems (white bars) at the four studied locations. CSYp *_i_*: yield potential of cropping system *i*. CSYa *_i_*: actual yield of cropping system *i*. CSYp^*^: cropping system yield potential. CSYp^*^ was calculated as CSYp of an alternative cropping system identified according to the framework in Fig. 2 and described in Table 4. See main text Section 2.1 for full definitions.

**Fig. 6:**
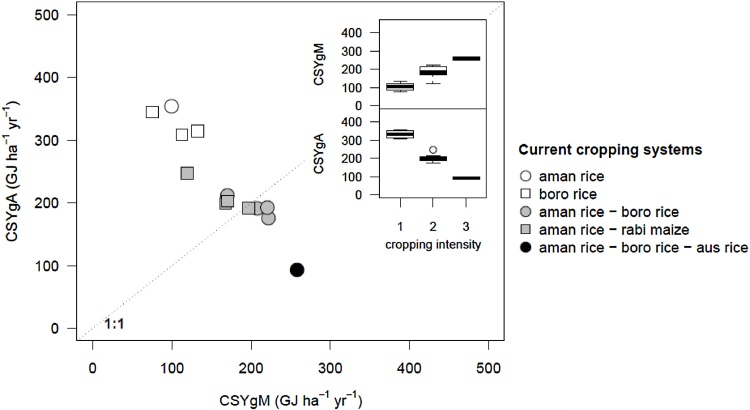
Components of the cropping system yield gaps. CSYgM: yield gap due to the temporal arrangement of crops. CSYgA: yield gap due to the management of individual crops within the current cropping system. See Fig. 1 and main text Section 2.1 for definitions. Note that dot color indicate cropping intensity (number of crops per year) of current cropping systems as follows: white (1), grey (2) and black (3). Inserts show relationships between components of the cropping system yield gap and cropping intensity of current cropping systems.

Smallest CSYg was found for the annual triple-rice system (*aman* rice – *boro* rice – *aus* rice) in Rajshahi (351 GJ ha^−1^ yr^−1^) while the largest one was found for the single *aman* rice CS in Bogra (454 GJ ha^−1^ yr^−1^) (Fig. 5). The CSYg components varied among current CSs: CSYgA (yield gap due to the spatial and/or temporal crop arrangement, which includes differences in cropping intensity between current CS and CS^*^) was higher than CSYgM (the yield gap due to the management of individual crops within the current system) in 8 out 13 of the CS × location combinations under study (Fig. 6). CSYgM increased with cropping intensity of current CSs and CSYgA decreased with cropping intensity of current CSs (see Fig. 6 inserts). Consequently, when cropping intensity is low, opportunities to increase yield from improved management of individual crops within current CSs are estimated to be lower than opportunities to increase yield from increased cropping intensity, and vice-versa. Interestingly, the triple rice CS in Rajshahi had a positive CSYgA, showing that even when cropping intensity is high (*i.e.*, three crops per year) there still appears to be room for improved timing of each crop cycle within the system.

As systems with different cropping intensities coexist in a given area, it is necessary to consider their relative proportions on a cropland area basis to estimate CSYg and its components at the required spatial scale. Lowest average CSYg was found in Rajshahi (383 GJ ha^−1^ yr^−1^) and highest CSYg was found in Dhaka (406 GJ ha^−1^ yr^−1^) (Table 5). Average CSYgA was higher than average CSYgM at all locations (Bogra, Dhaka, Rajshahi, and Rangpur), but a greater difference was observed in Dhaka and Rangpur where CSYgA was respectively 43% and 64% higher than CSYgM, as compared to Bogra and Rajshahi where CSYgA was only 28% and 26% higher than CSYgM, respectively. Therefore, improving the temporal arrangement of crops appeared to be more promising than improving the management of individual crops within current CSs at all locations, and this is especially true in Dhaka and Rangpur. These results are consistent with current regional cropping intensities which are lower in Dhaka and Rangpur than in Bogra and Rajshahi (Table 3), suggesting more room to increase cropping intensity in Bogra and Rangpur.

### 3.3 Resource requirements to achieve cropping system yield potential

Greater CSYp was associated with larger water and N requirements (Table 6). Given the high yield potential of alternative CS^*^ (>30 t ha^−1^ yr^−1^), the amount of water required to achieve CSYp^*^ was sizeable, at about 1500–1600 mm yr^−1^. This was greater than for current CSs, but still represented less than the annual amount of rainfall, except for the triple rice CS in Rajshahi. The annual crop N requirements of the different CS^*^ were very large, ranging from about 750 to 800 kg N ha^−1^ yr^−1^. CS^*^ were much more efficient at intercepting incoming PAR than current CSs as they intercepted 60–70% of annual incoming PAR while current CSs only intercepted 10–40% of it. It is also likely that greater use of mechanized planters and transplanters, and grain harvesters of appropriate size for small farms will be needed to facilitate more timely planting in CS^*^ that utilize later maturing crop varieties.

## 4 Discussion

Previous yield gap analyses focused on single crops and did not consider alternative systems involving new spatial and/or temporal arrangement of crops (Van Ittersum et al., 2013). In this paper, we propose a framework to identify alternative systems and evaluate them in comparison to existing ones. This framework allows determining the largest opportunities for yield increase: improving the spatial and/or temporal arrangement of crops, the management of current individual crops, or both. This is particularly relevant as areas suitable for double or triple cropping are increasing due to climate change in many environments like the Pampas in Argentina (Andrade et al., 2015), Spain (Meza et al., 2008), southern Great Plains in the U.S. (Seifert and Lobell, 2015), parts of China (Liu et al., 2013) and Tibetan Plateau (Zhang et al., 2013). Therefore, a key question is: how much extra-food could be produced from increasing the temporal cropping intensity and how it compares with closing yield gaps of individual crops? Our evaluation framework could answer this question at local, regional, national, and global scales when used in conjunction with a proper upscaling protocol (Van Bussel et al., 2015).

The Bangladesh case study showed that improving the spatial arrangement of crops can give higher productivity gains than improving the management of individual crops within current systems, which supports our working hypothesis (Introduction). Moreover, we identified two locations (Dhaka and Rangpur) where improving the crop sequence was a more promising option than in the two other locations (Bogra and Rajshahi). An *aman* rice – *rabi* maize – *kharif-I* maize system was identified as a potentially viable alternative system in Rajshahi. This is consistent with the rapid expansion of this system (Timsina et al., 2011). In Bogra, Dhaka, and Rangpur, another alternative system was identified in which maize is grown during the *kharif-II* instead of the *kharif-I* season: *kharif-II* maize – *rabi* maize – *aus* rice. According to Timsina et al. (2010) and Ali et al. (2009), maize is already grown during the *kharif-II* season in parts of Bangladesh, which supports our findings that the identified alternative CS is a promising option. Collectively, these results support the assessment based on the framework presented in Fig. 2 to identify interesting alternative CSs. Also, recognizing that identifying alternative systems is challenging due to the cost and time required to conduct exploratory field studies, our framework could add value to tools commonly used in the fields of cropping and farming system design (Malézieux, 2012, Martin et al., 2013), like crop sequence generators (Dogliotti et al., 2003), analysis of large farm surveys databases (Henderson et al., 2016, Farmaha et al., 2016), tracking on-farm innovations (Salembier et al., 2016), and participatory approaches (Le Bellec et al., 2012). Likewise, our framework could be expanded to consider other factors including requirements and availability of labor and capital, water and nutrients (see below), other production criteria (*e.g.* protein yield), and diseases and pests in multiple systems (Kirkegaard et al., 2008, Ratnadass et al., 2012). Therefore the work presented in this paper would be useful to bridge the gap between yield gap analysis and farming system design.

High-yielding CSs generally require large amounts of water and nutrients, which could increase the risk of adverse environmental effects such as groundwater depletion and nutrient losses *via* leaching and other pathways that have negative environmental impact. In this study, water and N requirements to achieve CSYp^*^ were estimated to be higher than 1500 mm yr^−1^ and 700 kg N ha^−1^ yr^−1^, respectively. Hence, assessment of CSYg based on the framework shown in Fig. 2 needs to be complemented with an evaluation of resource requirements and associated environmental footprint per unit of land and production (Grassini and Cassman, 2012). Our analysis considered N and water requirements and it is noteworthy that the evaluation framework provides underpinning data to evaluate these requirements. Assessment of resource requirements should be undertaken on an annual basis for consistency with the definition of CSYp^*^ and associated yield gap.

In this paper, the proposed framework has been applied to irrigated rice-maize systems in Bangladesh. We believe this framework could be applied to other CSs, be they rainfed or characterized by longer crop sequences as in temperate regions (*e.g.* north-west Europe). However, in rainfed systems, effects of previous crop(s) on residual soil water should be taken into account. Moreover, recent progress towards development of a theoretical framework for applying yield gap analysis to livestock systems (Van der Linden et al., 2015) suggest opportunities to expand yield gap analysis to crop-livestock systems, but this is beyond the scope of this paper.

In the real world, the definition of crop yield is not static. It has evolved on historical time scales from joules joule^−1^ for hunters-gatherers, to grains grain^−1^ at the early stages of agriculture to kg per unit land area in contemporary agriculture, as shown by Evans (1993). Evans also emphasized the need to include the time dimension in the definition of yield (kg ha^−1^ yr^−1^) to account for cropping intensity. From this historical perspective, the definitions and methods outlined in this paper are unlikely to be definitive; we rather expect our propositions to be improved in further work. We also expect our work will stimulate scientific activities in defining, evaluating and closing yield gaps at the cropping system level, an often overlooked aspect of food security.

## Figures and Tables

**Table 1:** Examples of trade-offs between cropping-system yield and yield of individual crops reported in the literature.

Cropping system	Climate	References
A – Sequential (crops are not grown simultaneously)		
wheat-maize	temperate (Argentina, New-Zealand)	Monzon et al. (2007); Fletcher et al., (2011)
wheat-soybean	temperate (USA)	Shapiro et al. (1992)
maize-maize	semi-arid (Chile)	Meza et al. (2008)
maize-soybean	temperate (Argentina)	Monzon et al. (2014)
rice-rice-rice	tropical (Asia)	Evans, (1993)

B – Simultaneous (crops are, at least partly, grown simultaneously)		
maize-barley	semi-arid (China, Gansu)	Li et al. (2011)
maize-wheat	semi-arid (China, Gansu)	Li et al. (2011)
maize-faba bean	semi-arid (China, Gansu)	Li et al. (2011)
maize-pigeon pea	sub-humid (Brazil)	Baldé et al. (2011)
maize-soybean	temperate (Argentina)	Monzon et al. (2014)
maize-cassava	tropical	Mutsaers et al. (1993)
maize-potato	tropical	Wu et al. (2012)
wheat-pea	temperate (Europe)	Bedoussac and Justes (2010a,b), Bedoussac et al. (2015)
vegetable-vegetable	–	Yildirim and Guvenc (2005)

**Table 2:** Assumptions for estimation of the cropping system yield potential and associated yield gap.

Assumptions
**Assumptions related to the cropping system yield potential definition**
1. Crops are grown with no limitation of water or nutrients and kept free of biotic stresses (weeds, pests, and diseases).
2. Higher incidence of biotic stresses when approaching the yield potential is not taken into account.
3. Greater environmental footprint due to greater input (*e.g.*, fertilizer, water) use required to achieve yield potential is not taken into account.
4. Extra labor requirements to achieve yield potential are not taken into account.
5. Possible diminishing economic returns to investment in extra inputs to achieve yield potential are not taken into account.

**Assumptions related to the cropping system yield gap estimation**
6. Diet preferences are not explicitly taken into account to evaluate alternative cropping system but they are partly accounted for by the market existence criterion.
7. Simulation of cropping system yield potential does not require consideration of effects of crop rotations (on soil resource and biology).

**Table 3:** Description of major cropping systems involving rice and/or maize in the four selected locations: crop sequence, actual yield, potential yield and% area under each cropping system. Average yield per location was calculated as the mean over all cropping systems weighted by the percent area under each cropping system. All cropping systems presented here are existing ones.

Cropping system	Bogra	Dhaka	Rajshahi	Rangpur
**% area under each cropping system**				
aman rice	25	–	–	–
boro rice	–	40	25	30
aman rice – boro rice	50	40	25	30
aman rice – boro rice – aus rice	–	–	25	–
aman rice – rabi maize	25	20	25	40
*Average cropping intensity per location*	*1.75*	*1.60*	*2.00*	*1.70*

**Actual yield (t** **ha**^**−1**^ **yr**^**−1**^**)**				
aman rice	2.2	–	–	–
boro rice	–	4.3	4.1	3.9
aman rice – boro rice	6.1	5.8	6.7	6.3
aman rice – boro rice – aus rice	–	–	8.9	–
aman rice – rabi maize	8.1	6.4	7.2	9.3
*Average actual yield per location*	*5.0*	*5.0*	*6.0*	*6.5*

**Potential yield (t** **ha**^**−1**^ **yr**^**−1**^**)**				
aman rice	9.1 (6)^a^	–	–	–
boro rice	–	12.1 (4)	9.3 (10)	13.1 (4)
aman rice – boro rice	21.5 (5)	20.2 (4)	18.5 (8)	21.6 (4)
aman rice – boro rice – aus rice	–	–	26.8 (7)	–
aman rice – rabi maize	20.5 (5)	20.2 (4)	21.2 (6)	20.2 (5)
*Average potential yield per location*	*18.2 (4)*	*17.0 (4)*	*19.0(5)*	*18.5 (4)*

a Values in parenthesis indicate the coefficient of variation in yield (%) over years.

**Table 4:** Simulated cropping system yield potential in Bogra, Dhaka, Rajshani, and Rangpur, Bangladesh. CS^*^ is a new cropping system, defined as the system with highest energy return per unit of land and time (Eq. 3). CSYp^*^: yield potential of CS^*^, also called cropping system yield potential. Yp: yield potential of individual crops. All yields were simulated under no limitation by water, nutrients, pests and diseases. Yields are expressed in t ha^−1^of grain at standard moisture contents and GJ ha^−1^ of energy content of grain. Start: sowing date (maize) or transplanting date (rice). End: maturity date. Total length: number of days between sowing or transplanting and simulated physiological maturity. Three cropping seasons were considered: the rainy season *kharif-II* or *aman* from June-July to September-October, the dry season *boro* or *rabi* from October-November to February-March, and the *kharif-I* or *aus* season from March-April to May-June.

CS^*^	CSYp^*^ (t ha^−1^ yr^−1^)	CSYp^*^ (GJ ha^−1^ yr^−1^)	Total length	Season	Crop	Variety	Yp (t ha^−1^)	Yp (GJ ha^−1^)	start	end
Bogra										
maize-maize-rice	33.0 (7.5)^a^	486	295	Kharif-II	maize	late	9.4	139	15-Jun	5-Sept
				Rabi	maize	late	16.2	240	1-Oct	6-Feb
				Aus	rice	short	7.4	107	3-Mar	25-May

Dhaka										
maize-maize-rice	32.9 (6.3)	483	303	Kharif-II	maize	late	9.0	133	1-Jun	24-Aug
				Rabi	maize	late	13.9	206	15-Sept	31-Dec
				Aus	rice	Int.	10.0	144	14-Jan	6-May

Rajshahi										
rice-maize-maize	32.5 (5.3)	480	295	Aman	rice	short	4.9	71	1-Jul	8-Sept
				Rabi	maize	late	17.4	258	1-Oct	20-Feb
				Kharif-I	maize	late	10.2	151	15-Mar	9-Jun

Rangpur										
maize-maize-rice	34.2 (4.5)	504	297	Kharif-II	maize	late	9.5	141	15-Jun	7-Sept
				Rabi	maize	late	18.0	266	1-Oct	21-Feb
				Aus	rice	extra-short	6.7	97	17-Mar	26-May

a Values in parenthesis indicate the coefficient of variation in yield (%) over years.

**Table 5:** Average cropping system yield gap (CSYg) and its components in Bogra, Dhaka, Rajshani, and Rangpur, Bangladesh. Yields are expressed in t ha^−1^of grain at standard moisture contents and GJ ha^−1^ of energy content of grain. To obtain a single estimate of CSYg and its components at each location, we weighted the CSYg of current CSs in that location by the proportional crop area devoted to each CS in the region surrounding that location. CSYgM: yield gap due to the management of current individual crops. CSYgA: yield gap due to spatial and/or temporal arrangement of crops. CSYa: actual yield of current cropping systems. CSYp: yield potential of current cropping systems. CSYp^*^: cropping system yield potential.

Metric	Bogra	Dhaka	Rajshahi	Rangpur	Bogra	Dhaka	Rajshahi	Rangpur
	t ha^−1^ yr^−1^				GJ ha^−1^ yr^−1^			
Yields								
CSYa	5.0	5.0	6.0	6.5	82	77	97	99
CSYp	18.2	17.0	19.0	18.5	259	244	266	252
CSYp^*^	33.0	32.5	32.9	34.2	486	483	480	504

Yield gaps								
CSYgM	13.2	12.0	13.0	12.0	177	167	169	153
CSYgA	14.8	15.5	13.9	15.7	227	239	214	252
CSYg	28.0	27.5	26.9	27.7	404	406	383	405

**Table 6:** Estimates of radiation, water and N capture at the cropping system level required to achieve potential yield for current and alternative cropping systems. For radiation and water, values were calculated by simulation under potential conditions (*i.e.* with no limitation by water, nutrients, pests and diseases). For N, values were calculated according to Eq. (6) and (7). PAR: photosynthetically active radiation.

Cropping system	Annual PAR	Annual PAR intercepted		Annual rainfall	Annual crop evapotranspiration		Annual crop N uptake
	MJ m^−2^ yr^−1^	MJ m^−2^ yr^−1^	% annual PAR		mm yr^−1^	% annual rainfall	kg N ha^−1^ yr^−1^
**Bogra**							
aman rice	6252	838	13	1767	560	32	276
aman rice – boro rice		1938	31		1279	72	748
aman rice – rabi maize		824	13		558	32	467
kharif-II maize – rabi maize – aus rice^a^		3844	61		1524	86	769

**Dhaka**							
boro rice	6145	1066	17	2050	666	32	452
aman rice – boro rice		1838	30		1202	59	677
aman rice – rabi maize		792	13		524	26	471
kharif-II maize – rabi maize – aus rice^a^		3662	60		1493	73	746

**Rajshahi**							
boro rice	6434	1069	17	1459	795	54	286
aman rice – boro rice		1925	30		1372	94	567
aman rice – boro rice – aus rice		2661	41		1854	127	805
aman rice – rabi maize		856	13		577	40	462
aman rice – rabi maize – kharif-I maize^a^		4443	69		1585	109	800

**Rangpur**							
boro rice	6340	1169	18	2303	759	33	521
aman rice – boro rice		1999	32		1345	58	773
aman rice – rabi maize		790	12		482	21	402
kharif-II maize – rabi maize – aus rice^a^		4117	65		1583	69	818

a Alternative cropping system (CS^*^) identified by simulation.

## Footnotes

1 Include all feasible agricultural land uses and practices, such as crops, pastures, livestock, fallow, green manure, and forestry.
