# Supplementary material for: Estimating yield gaps at the cropping system level
Source: Field Crops Res. 2017 May;206:21–32. doi: 10.1016/j.fcr.2017.02.008 (PMC5421155; doi:10.1016/j.fcr.2017.02.008)
Supplement: Supplementary file 1 [file mmc1.docx]

# Supplementary information

**Table S1.Dominant current cropping systems involving maize, rice and wheat in Bogra, Dhaka, Rajshahi, and Rangpur according to the Global Yield Gap Atlas (**[**www.yieldgap.org**](http://www.yieldgap.org)**).** Three cropping seasons were considered: the rainy season *kharif-II* or *aman* from June-July to September-October, the dry season *boro* or *rabi* from October-November to February-March, and the *kharif-I* or *aus* season from March-April to May-June.

| **Location name and major cropping systems** | |
| --- | --- |
| **Bogra** | **Dhaka** |
| Aman rice  Aman rice – boro rice  Aman rice – potato – rabi maize  Aman rice – potato – boro rice  Aman rice – wheat - mungbean | Rabi maize – jute  Aman rice – boro rice  Wheat – jute  Rabi maize – jute – aman rice  Aman rice – wheat – jute  Vegetable – wheat – jute – vegetable |
| **Rajshahi** | **Rangpur** |
| Aman rice – rabi maize  Aman rice – boro rice  Aman rice – potato – aus rice  Aman rice – boro rice – aus rice  Aman rice – wheat - mungbean | Aman rice – wheat  Aman rice – rabi maize  Aman rice – potato – rabi maize  Aman rice – potato – boro rice |

**Table S2. Sowing (maize) and transplanting (rice) dates of dominant current cropping systems involving rice and/or maize in Bogra, Dhaka, Rajshahi, and Rangpur.** Three cropping seasons were considered: the rainy season *kharif-II* or *aman* from June-July to September-October, the dry season *boro* or *rabi* from October-November to February-March, and the *kharif-I* or *aus* season from March-April to May-June. *Source: Global Yield Gap Atlas* ([www.yieldgap.org](http://www.yieldgap.org)).

| **Cropping system** | **Crop** | **Bogra** | **Dhaka** | **Rajshahi** | **Rangpur** |
| --- | --- | --- | --- | --- | --- |
| aman rice | aman rice | 12-Aug | - | - | - |
| boro rice | boro rice | - | 25-Dec | 14-Jan | 14-Jan |
| aman rice – boro rice | aman rice | 12-Aug | 2-Aug | 4-Aug | 15-Jul |
|  | boro rice | 10-Jan | 1-Jan | 14-Jan | 1-Jan |
| aman rice – boro rice – aus rice | aman rice | - | - | 13-Aug | - |
|  | boro rice | - | - | 19-Dec | - |
|  | aus rice | - | - | 2-May | - |
| aman rice – rabi maize | aman rice | 4-Aug | 2-Aug | 4-Aug | 15-Jul |
|  | rabi maize | 5-Feb | 1-Nov | 20-Nov | 1-Jan |

**Table S3. Phenology parameters of the 4 varieties used in the simulations of rice and maize.** DVRJ: Developmental rate during juvenile phase. DVRI: Developmental rate during photoperiod sensitive phase. DVRP: Developmental rate during panicle development phase. DVRR: Developmental rate during reproductive phase. Maturity days: average simulated number of days from sowing/transplanting to physiological maturity. GDD: Growing Degree Days. For rice, developmental rates come from Timsina et al. (2010, 2011). In this work, coefficients for an intermediate maturity type as used for IR72 were adapted from Bouman et al. (2001). For maize, GDD to maturity correspond to cultivars available in the market (see for example: <http://www.syngenta-us.com/corn/nk/product-finder>).

|  | **Rice -** ORYZA | | | | |  | | **Maize –** Hybrid Maize | | |
| --- | --- | --- | --- | --- | --- | --- | --- | --- | --- | --- |
| **Variety** | **DVRJ** | **DVRI** | **DVRP** | **DVRR** | **Maturity (days)** |  | **GDD to maturity (°Cd)** | | **Maturity (days)** |  |
| extra-short | .000883 | .000758 | .001400 | .002600 | 70 |  | 1000 | | 70 |  |
| short | .000783 | .000758 | .001000 | .002300 | 85 |  | 1200 | | 80 |  |
| intermediate | .000683 | .000758 | .000749 | .001900 | 100 |  | 1400 | | 90 |  |
| late | .000583 | .000758 | .000549 | .001600 | 125 |  | 1600 | | 105 |  |

**Table S4. Actual yields and potential yields, and crop N uptake required to achieve yield potential of individual crops of current rice and rice-maize cropping systems in the four selected locations.** Actual and potential yields were retrieved from the Global Yield Gap Atlas ([www.yieldgap.org](http://www.yieldgap.org)). Crop N uptake required to achieve the yield potential was calculated as a function of crop yield as described in Cassman *et al.* (2002) and in Eq. 6 and 7.

| **Cropping system** | **Crop** | **Actual yield**  **(t ha^-1^)** | **Yield potential (t ha^-1^)** | **Crop N uptake**  **(kgN ha^-1^)** |
| --- | --- | --- | --- | --- |
| **Bogra** |  |  |  |  |
| aman rice | aman rice | 2.2 | 9.1 | 276 |
| aman rice – boro rice | aman rice | 2.2 | 9.1 | 276 |
|  | boro rice | 3.9 | 12.4 | 472 |
| aman rice – rabi maize | aman rice | 2.2 | 8.5 | 245 |
|  | rabi maize | 5.9 | 11.1 | 222 |
| **Dhaka** |  |  |  |  |
| boro rice | boro rice | 4.3 | 12.1 | 452 |
| aman rice – boro rice | aman rice | 1.5 | 8.2 | 231 |
|  | boro rice | 4.3 | 12.0 | 446 |
| aman rice – rabi maize | aman rice | 1.5 | 8.2 | 231 |
|  | rabi maize | 4.9 | 11.7 | 240 |
| **Rajshahi** |  |  |  |  |
| boro rice | boro rice | 4.1 | 9.3 | 286 |
| aman rice – boro rice | aman rice | 2.6 | 9.2 | 281 |
|  | boro rice | 4.1 | 9.3 | 286 |
| aman rice – boro rice – aus rice | aman rice | 2.6 | 9.9 | 318 |
|  | boro rice | 4.1 | 8.0 | 222 |
|  | aus rice | 2.2 | 8.9 | 265 |
| aman rice – rabi maize | aman rice | 2.6 | 9.2 | 281 |
|  | rabi maize | 4.6 | 9.7 | 181 |
| **Rangpur** |  |  |  |  |
| boro rice | boro rice | 3.9 | 13.1 | 521 |
| aman rice – boro rice | aman rice | 2.4 | 8.2 | 231 |
|  | boro rice | 3.9 | 13.4 | 542 |
| aman rice – rabi maize | aman rice | 2.4 | 8.2 | 231 |
|  | rabi maize | 6.9 | 9.3 | 171 |

**Table S5. Actual yields in Bangladesh.** Yields are in t ha^-1^.*Source: Bangladesh Bureau of Statistics.*

| **District** | **crop** | **year** | **yield** | **District** | **crop** | **year** | **yield** |
| --- | --- | --- | --- | --- | --- | --- | --- |
| Bogra | aman rice | 2008 | 2.20 | Bogra | boro rice | 2008 | 4.00 |
| Dhaka | aman rice | 2008 | 1.31 | Dhaka | boro rice | 2008 | 4.43 |
| Rajshahi | aman rice | 2008 | 2.57 | Rajshahi | boro rice | 2008 | 4.36 |
| Rangpur | aman rice | 2008 | 1.97 | Rangpur | boro rice | 2008 | 3.90 |
| Bogra | aman rice | 2009 | 2.15 | Bogra | boro rice | 2009 | 4.15 |
| Dhaka | aman rice | 2009 | 1.78 | Dhaka | boro rice | 2009 | 4.28 |
| Rajshahi | aman rice | 2009 | 2.59 | Rajshahi | boro rice | 2009 | 4.12 |
| Rangpur | aman rice | 2009 | 2.28 | Rangpur | boro rice | 2009 | 3.93 |
| Bogra | aman rice | 2010 | 2.05 | Bogra | boro rice | 2010 | 3.99 |
| Dhaka | aman rice | 2010 | 1.42 | Dhaka | boro rice | 2010 | 4.02 |
| Rajshahi | aman rice | 2010 | 2.56 | Rajshahi | boro rice | 2010 | 3.83 |
| Rangpur | aman rice | 2010 | 2.39 | Rangpur | boro rice | 2010 | 4.05 |
| Bogra | aman rice | 2011 | 2.16 | Bogra | boro rice | 2011 | 3.73 |
| Dhaka | aman rice | 2011 | 1.44 | Dhaka | boro rice | 2011 | 4.44 |
| Rajshahi | aman rice | 2011 | 2.67 | Rajshahi | boro rice | 2011 | 4.01 |
| Rangpur | aman rice | 2011 | 2.65 | Rangpur | boro rice | 2011 | 3.76 |
| Bogra | aman rice | 2012 | 2.34 | Bogra | boro rice | 2012 | 3.74 |
| Dhaka | aman rice | 2012 | 1.50 | Dhaka | boro rice | 2012 | 4.20 |
| Rajshahi | aman rice | 2012 | 2.54 | Rajshahi | boro rice | 2012 | 4.07 |
| Rangpur | aman rice | 2012 | 2.67 | Rangpur | boro rice | 2012 | 3.76 |
| Bogra | aus rice | 2008 | 2.09 | Dhaka | maize | 2008 | 5.03 |
| Dhaka | aus rice | 2008 | 1.01 | Bogra | maize | 2008 | 6.78 |
| Rajshahi | aus rice | 2008 | 1.98 | Rajshahi | maize | 2008 | 4.81 |
| Rangpur | aus rice | 2008 | 0.00 | Rangpur | maize | 2008 | 6.98 |
| Bogra | aus rice | 2009 | 2.07 | Dhaka | maize | 2009 | 5.48 |
| Dhaka | aus rice | 2009 | 1.33 | Bogra | maize | 2009 | 5.81 |
| Rajshahi | aus rice | 2009 | 2.31 | Rajshahi | maize | 2009 | 5.08 |
| Rangpur | aus rice | 2009 | 0.00 | Rangpur | maize | 2009 | 7.64 |
| Bogra | aus rice | 2010 | 1.69 | Dhaka | maize | 2010 | 4.47 |
| Dhaka | aus rice | 2010 | 1.50 | Bogra | maize | 2010 | 5.61 |
| Rajshahi | aus rice | 2010 | 2.06 | Rajshahi | maize | 2010 | 4.47 |
| Rangpur | aus rice | 2010 | 0.00 | Rangpur | maize | 2010 | 6.62 |
| Bogra | aus rice | 2011 | 1.96 | Dhaka | maize | 2011 | 4.56 |
| Dhaka | aus rice | 2011 | 1.40 | Bogra | maize | 2011 | 5.88 |
| Rajshahi | aus rice | 2011 | 2.16 | Rajshahi | maize | 2011 | 4.36 |
| Rangpur | aus rice | 2011 | 0.00 | Rangpur | maize | 2011 | 6.28 |
| Bogra | aus rice | 2012 | 2.08 | Dhaka | maize | 2012 | 5.09 |
| Dhaka | aus rice | 2012 | 1.48 | Bogra | maize | 2012 | 5.55 |
| Rajshahi | aus rice | 2012 | 2.44 | Rajshahi | maize | 2012 | 4.47 |
| Rangpur | aus rice | 2012 | 0.00 | Rangpur | maize | 2012 | 6.99 |

**Figure S1. Monthly average incoming solar radiation, maximum (Tmax) and minimum temperature (Tmin), total rainfall, and total reference evapotranspiration (ETo) based on long-term (1992-2005) weather data in Bogra, Dhaka, Rajshahi and Rangpur (Bangladesh).** Fig. 3 maps these locations. Average total annual rainfall and reference evapotranspiration are shown in upper panels. Dotted lines in the top left panel denote the three main cropping seasons (*aman or kharif-II*, *boro or rabi, aus or kharif-I*). *Source: Global Yield Gap Atlas (www.yieldgap.org).*

**Figure S2. Simulated variability in yield potential (over 1992-2005) of rice (top row) and maize (bottom row) crops in four locations as related to crop phenology and sowing (for maize) or transplanting (for rice) date.** Variability in potential yield is expressed as the coefficient of variation (CV, %) in yield potential over time. DOY: day of year.


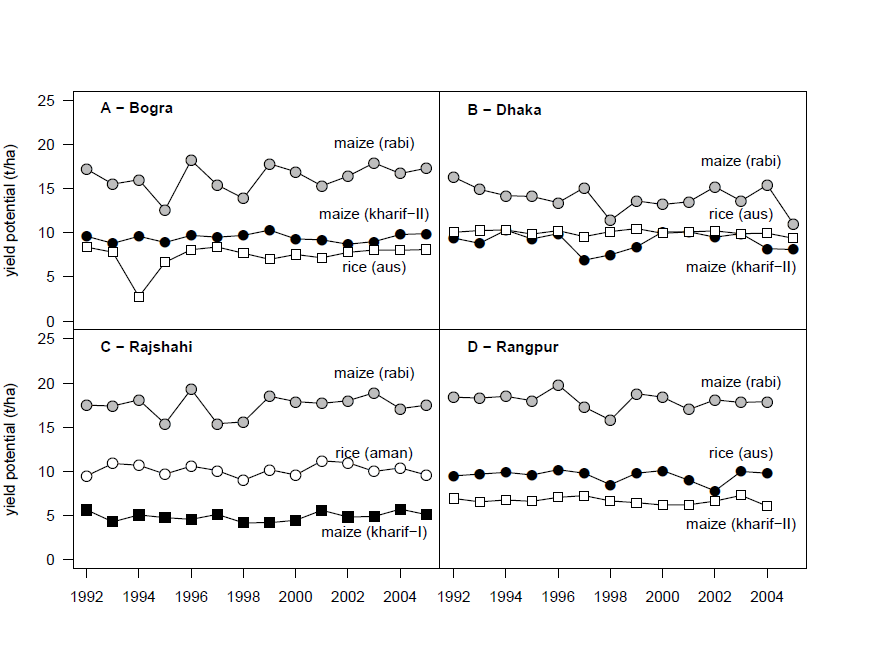


**Figure S3. Simulated yield potential of individual crops in alternative cropping systems (CS^*^) in (A) Bogra, (B) Dhaka, (C) Rajshahi, and (D) Rangpur in Bangladesh.** CS^*^ is a new cropping system, defined as the system with highest energy return per unit of land and time (Eq. 3) and identified following the framework in Fig. 2. Words in parentheses refer to the three main cropping seasons in Bangladesh: *kharif-II* or *aman* (from June-July to Sept-Oct) which is the rainy season, *boro* or *rabi* (from Oct-Nov to Feb-Mar) which is the dry season and *aus* or *kharif-I* (from Mar-Apr to May-June).
